# Post-concussive mTBI in Student Athletes: MRI Features and Machine Learning

**DOI:** 10.3389/fneur.2021.734329

**Published:** 2022-01-10

**Authors:** José Tamez-Peña, Peter Rosella, Saara Totterman, Edward Schreyer, Patricia Gonzalez, Arun Venkataraman, Steven P. Meyers

**Affiliations:** ^1^Tecnologico de Monterrey, Escuela de Medicina, Monterrey, Mexico; ^2^Qmetrics Technologies, Rochester, NY, United States; ^3^UR Imaging-UMI, University of Rochester Medical Center, University of Rochester, Rochester, NY, United States

**Keywords:** mTBI, post-concussion syndrome, quantitative MRI, diffusion imaging, radiomics

## Abstract

**Purpose:** To determine and characterize the radiomics features from structural MRI (MPRAGE) and Diffusion Tensor Imaging (DTI) associated with the presence of mild traumatic brain injuries on student athletes with post-concussive syndrome (PCS).

**Material and Methods:** 122 student athletes (65 M, 57 F), median (IQR) age 18.8 (15–20) years, with a mixed level of play and sports activities, with a known history of concussion and clinical PCS, and 27 (15 M, 12 F), median (IQR) age 20 (19, 21) years, concussion free athlete subjects were MRI imaged in a clinical MR machine. MPRAGE and DTI-FA and DTI-ADC images were used to extract radiomic features from white and gray matter regions within the entire brain (2 ROI) and the eight main lobes of the brain (16 ROI) for a total of 18 analyzed regions. Radiomic features were divided into five different data sets used to train and cross-validate five different filter-based Support Vector Machines. The top selected features of the top model were described. Furthermore, the test predictions of the top four models were ensembled into a single average prediction. The average prediction was evaluated for the association to the number of concussions and time from injury.

**Results:** Ninety-one PCS subjects passed inclusion criteria (91 Cases, 27 controls). The average prediction of the top four models had a sensitivity of 0.80, 95% CI: [0.71, 0.88] and specificity of 0.74 95%CI [0.54, 0.89] for distinguishing subjects from controls. The white matter features were strongly associated with mTBI, while the whole-brain analysis of gray matter showed the worst association. The predictive index was significantly associated with the number of concussions (*p* < 0.0001) and associated with the time from injury (*p* < 0.01).

**Conclusion:** MRI Radiomic features are associated with a history of mTBI and they were successfully used to build a predictive machine learning model for mTBI for subjects with PCS associated with a history of one or more concussions.

## Introduction

Mild traumatic brain injuries (mTBI) or “Concussion” with Glascow Coma Scale scores of 13 or greater result in transient alterations in brain function with or without loss of consciousness (1). It has been estimated that sports and other recreational activities result in between 1.6 and 3.8 million concussions annually in the U.S. (2). Moreover, 15% of mTBI patients suffer from post-concussion syndrome (PCS) characterized by delayed recovery after 3–4 weeks with physical, cognitive, and emotional complaints including headaches, poor sleep, poor concentration, dizziness, and irritability (3, 4). Furthermore, it has been reported that concussed subjects are at three times higher risk of depression even decades after the injury (5) with characteristic imaging patterns of white matter tracts (6). On the other hand, the recovery time of sports-related concussion (SRC) for adolescents takes 3–4 weeks, longer than the commonly reported 7–14 days for adults (7, 8).

Routine clinical MRI studies do not show abnormalities in most SRC and PCS patients, however, diffusion-weighted (DWI) and diffusion tensor imaging (DTI) have shown variable localized, diffuse, and widespread changes after mild injury (9, 10). Prior concussions lengthened the normalization of changes and made the athletes more vulnerable to recurrent concussions (11). Further, the degree and extent of the acute post-injury changes were associated with clinical outcomes and delayed recovery. Results from longitudinal studies have shown that the diffusivity changes lasted (“past symptom resolution”) beyond the point when athletes were asymptomatic and were medically cleared to return to play, with abnormal diffusion parameters lasting several months to over a year after the injury (10).

At present, many research programs are trying to use AI including deep and machine learning, which have been applied to medical image data in other neurological diseases, to develop validated clinical image-based tools to identify post-concussive changes, to correlate them to clinical symptoms, and to predict recovery time and clinical outcomes (12, 13). Several studies used ML to identify parameters from multi-parametric diffusion and structural MR image data to best discriminate and characterize mTBI subjects (13). Although their results were promising, so far there is no clinical tool or method which would indicate if PCS patients sustained a structural brain injury or determine its location or extent based on MRI images. Tissue texture analysis which evaluates the 3D signal intensity behavior of medical images was applied on brain MRI in other brain diseases (14). A few reports have used 3D structural and diffusivity texture features to evaluate changes in SRC and subsequent PCS (10).

Here we evaluated whether radiomics and ML can be used to differentiate patients that suffered mTBI with subsequent post-concussion syndrome from concussion-free athletes. To achieve the aim of this study, a small set of student athletes with SRC-related post-concussion syndrome were recruited, as well as a small set of concussion-free student athletes as controls. The data population was studied with state-of-the-art clinical MRI pulse sequences, quantitative image analysis, descriptive statistical procedures, and machine learning (ML) models that enabled the detection of PCS in affected subjects. Finally, we examined the longitudinal behavior of the predictive index and its association with the number of concussions.

## Materials and Methods

### Participants

**Figure 1:**
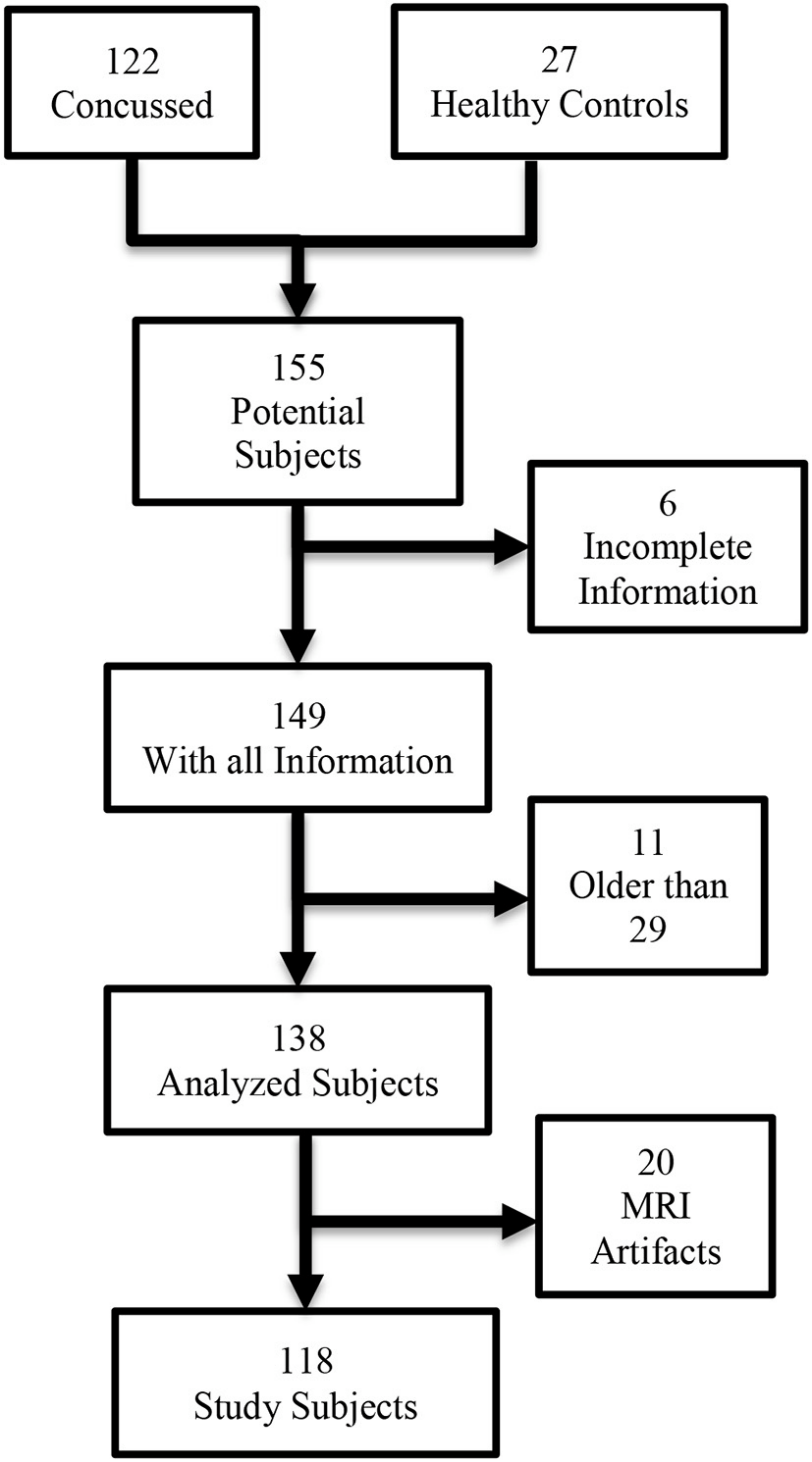
Inclusion criteria for post-concussive syndrome radiomics association study.

This retrospective analysis was approved by the Research Subjects Review Board of the University of Rochester. The study consisted of 122 subjects with a mixed level of play and sports activities. The concussed subjects were 65 males and 57 females, with a history of concussion who were High School or College Athletes with a history of one or more concussions and post-concussive symptoms who were diagnosed and evaluated by Sports Medicine Physicians; Physical Medicine, and Rehabilitation Physicians, or Neurologists. These patients were referred for MRI because of persistent clinical symptoms after mTBI from 2016 to 2019. The median (IQR) age for both male and female athletes was 17 (15–20) years. All case subjects in our study were referred to the University outpatient imaging center and imaged on the same 3T MRI scanner due to incomplete resolution of symptoms after concussion. Inclusion criteria included a history of concussion and PCS, while exclusion criteria included dental braces, prior brain surgery, ventricular shunt, skull fractures, or standard contraindications for MR imaging. Diagnosis of concussion was made by ED physicians initially and PCS was determined by neurologists, physical medicine and rehabilitation physicians, and/or sports medicine physicians. The number of previous concussions, loss of consciousness (LOC) at the most recent concussion, and time between injury and MRI were extracted by a clinician from electronic medical records. All evaluations included the Acute Concussion Evaluation (ACE) (15) as well as Post-concussion Symptom Scores (PSS) (16). The control group consisted of 27 normal student athletes, 15 males and 12 females with no history of head trauma and a median (IQR) age of 20 (19–21). In addition to the number of concussions, and PSS, each patient was characterized by gender, age, weight, height, and the number of days elapsed from injury to the MRI examination. The case to control ratio was large in MRI subjects (Ratio = 4.5). Hence, we mitigated this ratio by selecting cases closer to the control group. All older college students (Age > 29) were removed, as well as all subjects with abnormal imaging findings evident on the conventional MRI pulse sequences. Figure 1 shows the specific inclusion-exclusion chart of the analyzed subjects.

### Image Acquisition

All MRI Exams for the concussed patients and control volunteers were performed between February 17, 2016, and May 14, 2019, on the same 3T Siemens Skyra MRI Scanner using a 20 channel Head/Neck Coil using the following imaging protocol: T1-MPRAGE (208 slices; 1x1x1 mm, TR = 1,200 ms, TE = 2.29 ms, TI = 600 ms), FOV = 250 mm, Flip angle = 8 degrees, 3D AXIAL SWI (88 slices/1.5 mm slice thickness (interleaved)/FOV 220 mm, TR 27 ms, TE 29 ms, 1 average), DOUBLE IR (FLAIR)-Fat-sat FLAIR 120 slices, 1.4 mm slice thickness, FOV 260 mm, TR 7,500 ms, TE 321 ms, TI 1: 3,000 ms; TI 2: 450 ms, 1 average, Acceleration factor 2, ref lines 24, Turbo Factor 256.

DTI acquisition parameters were AXIAL DTI/ TA: 10:14 min,70 slices, FOV 256, 2 mm slice thickness, TR 9,000 ms, TE 88 ms, Flip Angle 15°, 1 average, Acceleration Factor 2/ref lines 24, Diffusion directions 64, b-value 1: 0 s/mm^2^; b-value 2: 1,000 s/mm^2^ GRE Field Mapping for geometric and eddy current corrections 86 slices, FOV 256 mm, 2 mm slice thickness, TR 838 ms, TE1: 4.92 sec, TE2: 7.38 ms, Flip angle: 60°. All image sets used in this study were anonymized.

### Image Analysis

#### Image Preprocessing and Segmentation

**Figure 2:**
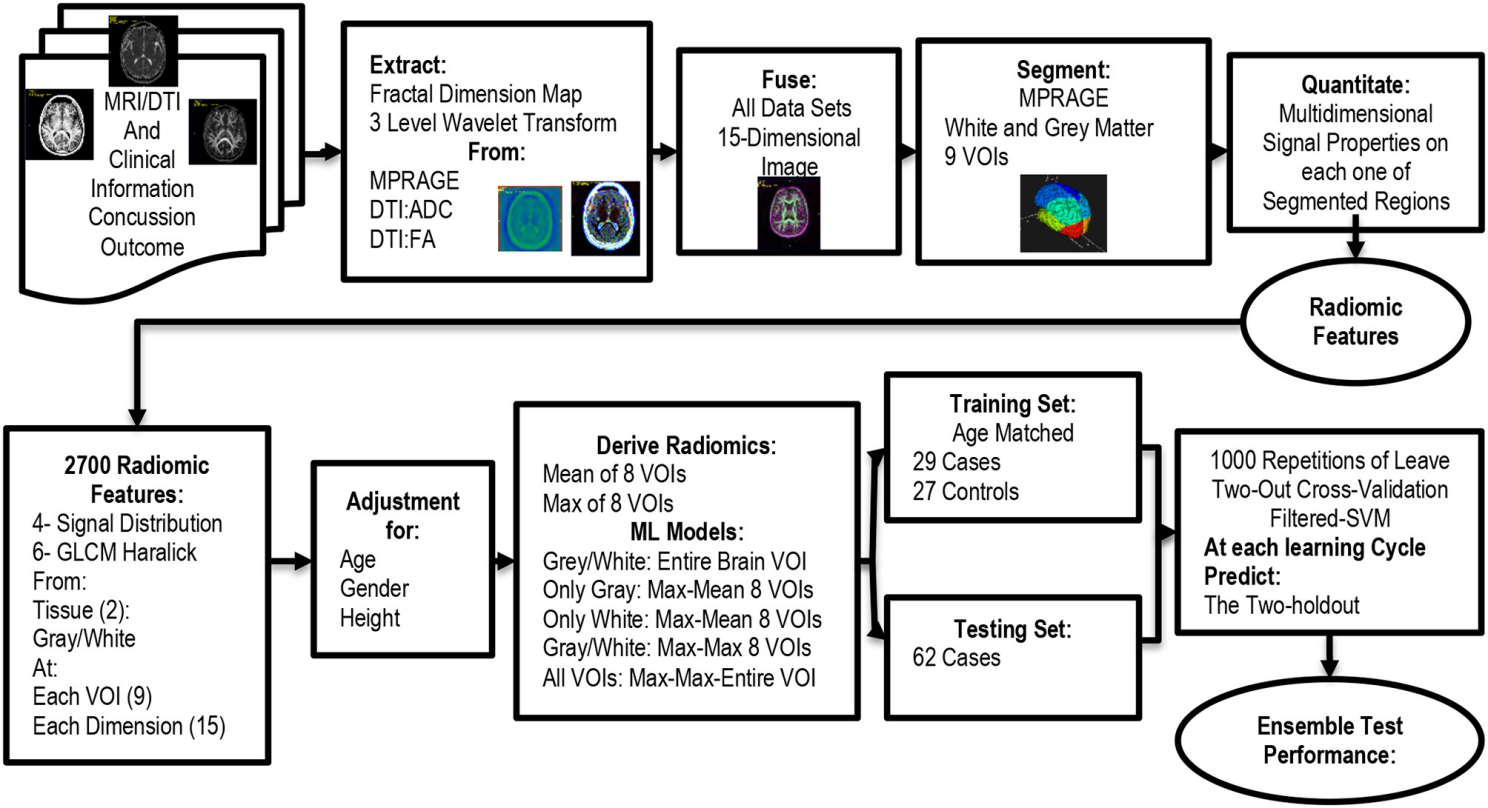
Flowchart for the analysis of the procedure used for the association analysis.

Figure 2 shows an overview of the image processing pipeline used for this study. The 64 gradients of the DTI acquisition were processed to estimate the Fractional Anisotropy Map (DTI-FA) and the Apparent Diffusion Coefficient (DTI-ADC). After that, the MPRAGE, DTI-FA, and DTI-ADC were preprocessed to extract the local fractal dimension (LFD) map and 3 levels wavelet decomposition (WD) of each volume data. The LFD algorithm estimates an index of the image texture complexity and it is done by analyzing the relative change in signal surface area at three different resolutions using the triangular prism surface area methods (17, 18). The three-dimensional (3D) WD was used to extract texture patterns from the imaging volume. At each decomposition level, the one-dimensional wavelet transform is applied sequentially on each one of the three spatial dimensions. This results in seven directional decompositions: High-High-High (HHH), High-High-Low (HHL), High-Low-High (HLH), High-Low-Low (HLL), Low-High-High (LHH), Low-High-Low (LHL), and Low-Low-High (LLH), and a scaled Low-Low-Low (LLL) decomposition. The wavelet process is repeated on each one of the LLL decompositions. In this study, we used Haar wavelets and we only extracted the first three levels of the WD (19). To reduce the by-voxel dimensions we computed the magnitude of the directional wavelets:


(1) $$\begin{array}{l} |WD{|}_{i}\\ \;=\;\sqrt{{\left(HHH_{i}\right)}^{2}+{\left(HHL_{i}\right)}^{2}+{\left(HLH_{i}\right)}^{2}+{\left(HLL_{i}\right)}^{2}+{\left(LHH_{i}\right)}^{2}+{\left(LHL_{i}\right)}^{2}+{\left(LLH_{i}\right)}^{2}} \end{array}$$

**Figure 3:**
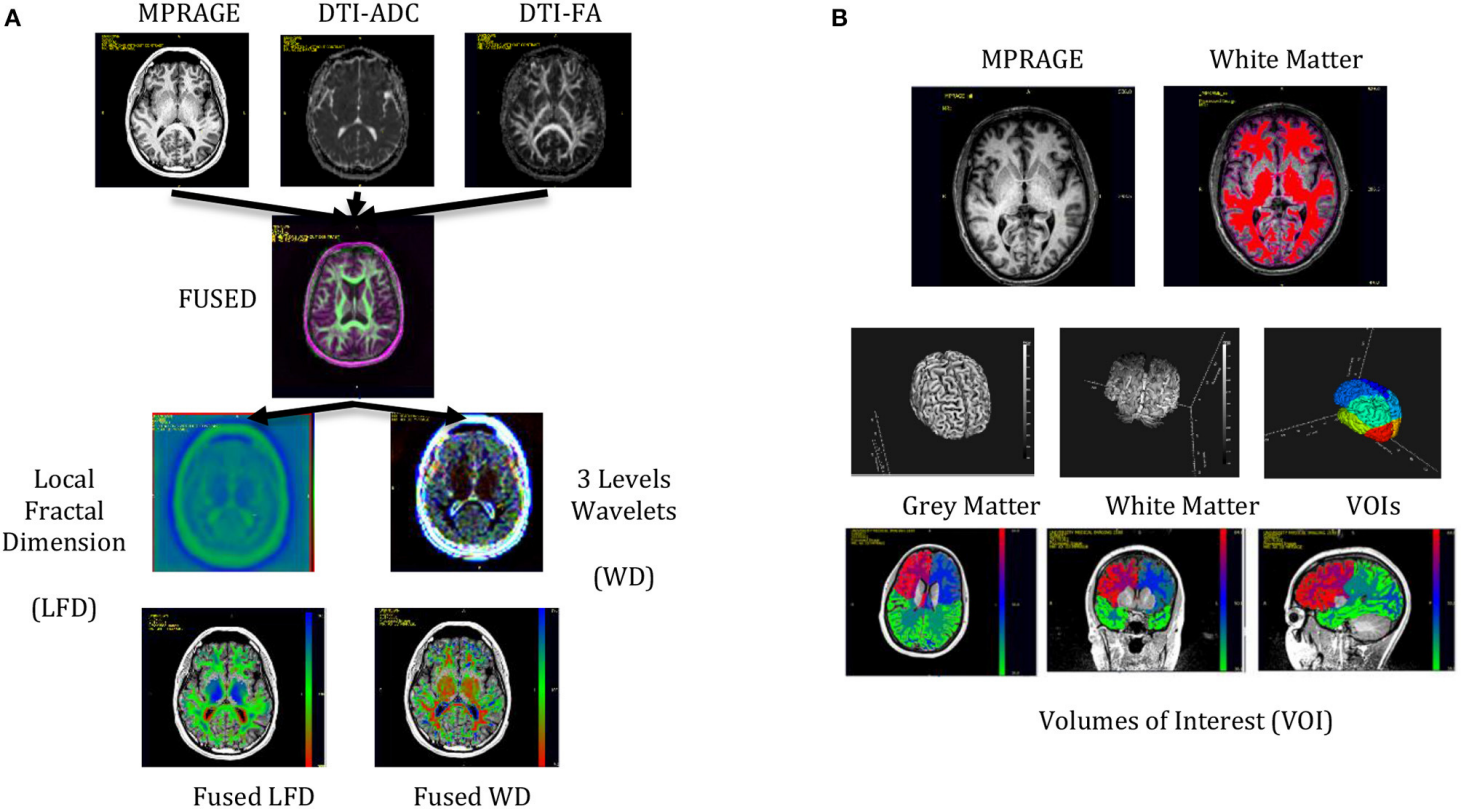
MRI data and Image processing steps carried out for the qMRI to PCS association. **(A)** Three MRI pulse sequences were fused and processed for the extraction of the local fractal dimension and the wavelets transforms. **(B)** The fused data sets were segmented into gray and white matter and subdivided in 8 volumes of interest. The two tissue types in each VOI plus the entire white and white matter created 18 regions of interest (ROI).

At each one of *i* = {1, 2, 3} decomposition levels. The LFD and the three |*WD*|_*i*_ were fused into a single 5-Dimensional MRI set for MPRAGE, DTI-FA, and DTI-ADC, hence creating a 3D volume where each voxel was described by 15 signal/textural features; i.e., signal dimensions. Once preprocessed, we proceeded to do the image segmentation. The segmentation process consisted of using the ICBM 152 Linear atlas and main lobe labels as a reference (20). The atlas defines the gray matter, white matter, and CSF tissues and it is divided by the volume of interest (VOI). In this study, we used only four lobes of the brain: Frontal, Parietal, Occipital, and Temporal for the left and right brain hemispheres, as seen in Figure 3. Furthermore, we only kept the definition of gray matter and white matter tissues. The modified 152c brain atlas, along with the T1 weighted MRI scans were used to segment each one of the MPRAGE MRI scans of the patients. This process created 8 VOIs and each VOI contained two tissues: gray and white matter. In other words, each brain was described by 16 different regions of interest (ROI) plus the entire white and gray matter for a total of 18 ROI per subject.

Once we segmented each MPRAGE image, we proceed to fuse all the data sets into a labeled 15-dimensional data set, that was fed into the quantification engine. Figure 3 shows a representation of the preprocessing and segmentation steps. All preprocessing and segmentation steps were carried out using CiPAS software (Qmetrics Technologies Rochester-NY).

#### Image Quantification

We computed radiomic features for each one of the dimensions of the 15-dimensional volume. The radiomic features consisted of four first-order features and GLCM features (21). The first other features were extracted from the histogram of the signal at each ROI: Mean, Standard Deviation, Skewness, and Entropy. The second-order features were extracted from the Gray Level Co-occurrence Matrix (GLCM): Contrast, Energy, Correlation, Homogeneity, Entropy Dissimilarity, and Mutual Information. We computed the GLCM using the four main directions and isotropic 2.0 × 2.0 × 2.0 mm^3^ voxels (21).

#### Quality Control Procedures

After the quantitation pipeline, each one of the MRI scans was reviewed by an experienced neuroradiologist (SM) for any artifacts that might affect the quality of the study, as well as for the presence of recent or remote intracranial hemorrhage, signal abnormalities in the brain, hydrocephalus, congenital or developmental anomalies. Furthermore, each one of them automatically segmented brain VOIs, and tissues were visually inspected to discard subjects with segmentation errors. Subjects with unknown concussions or injury time were also removed from this study.

### Machine Learning

The 10 image features extracted from the 18 ROI and 15 signal dimensions created a 2,700 (10 × 18 × 15) feature vector to be analyzed at the Machine Learning stage. A knowledge-based feature reduction strategy was used to decrease the dimensionality of the quantitative set. Studies have shown that mTBI mainly affects white matter tissue (22). Furthermore, it was assumed that the location of the concussion may have different effects on different areas of the brain, in other words, brain abnormalities associated with the concussion most probably are focal and only affect some of the 8 VOIs. The location of the concussion is unknown for this set of patients; hence, we hypothesized that the location of the injured tissue was associated with a large deviation from normal tissue behavior. Based on this hypothesis we computed the maximum deviation (Max-deviation) from the eight ROI means for each one of the 10 quantified signal features. To mitigate biases due to the presence of a single ROI abnormality, we used the trimmed mean as the estimator of the mean ($\left(\hat{Feature}\right)$. The Max deviation was computed as follows:


(2) $$\begin{array}{r} \text{Max}-\text{deviation}=\;max_{i\in VOI}|Feature_{i}-\hat{Feature}|, \end{array}$$

where


(3) $$\begin{array}{l} \hat{Feature}\\ \;=\frac{1}{6}([\sum_{i\in VOI}Feature_{i}]\;-\max\left(Feature_{i}\right)-min(Feature_{i})) \end{array}$$

To explore the origin of the signal associated with mTBI, we divided the entire feature set into five different feature sets. Table 1 shows the five-set used in this study. Furthermore, to mitigate training biases, we aimed to train models with an age-matched control to the case training set. Hence only case subjects within the age distribution of the control subjects were part of the training set. All case subjects not in the training set were part of the testing set.

**Table 1:** The five sets of features.

**Set**	**Name**	**Involved tissues**	**Volume of interest**	**Feature set**	**Number of explored features**
1	Mean white/gray matter	White and gray	Entire brain	Textural features	300
2	Max ROI Gray Matter	Gray	Eight VOI	Mean textural features, maximum of the eight	300
3	Max ROI white matter	White	Eight VOI	Mean textural features, maximum of the eight	300
4	Max ROI white/gray	White and gray	Eight VOI	Maximum textural features for white and gray	300
5	Mean plus max ROI white/gray	White and gray	Eight VOI	Mean textural features for both gray and white plus the maximum value	600

Each one of the five feature sets was modeled by a support vector machine (SVM) using the radial basis kernel function (23), with default initial hyper-parameters [γ = 1/(300 or 600), coef0 = 0, cost = 1, and ν = 0.5], and no hyper-parameters optimization. The features used by the SVM were selected by a feature selection process that ensembled the results of FDR (24) adjusted KS test, Wilcox text, *t*-student test, and the Minimum Redundancy Maximum Relevance (mRMR) (25). The training sets used for cross-validation were randomly selected by a two-hold out strategy that was repeated 1,000 times. At each stage of the cross-validation, one case sample and one control sample were a holdout sample and added to the testing set. The subjects that were not included in the training set were predicted at each one of the cross-validation steps. All the machine learning steps were done in R 4.0.3 using the FRESA.CAD 3.3.0 R package and the SVM function from the e1071 1.7-6 R package.

### Statistical Analysis Procedures

Due to the associations of brain structures with development age, body height, and weight (26), we adjusted the extracted features for age, weight, and height associations. The adjustments were estimated using subjects from a reference group and computed on all study subjects. Our study lacked a healthy reference group, hence we built a reference group using the imaged patients. Our reference group consisted of all control subjects plus a selected set of case subjects with only one concussion and more than 90 days from injury time. The rationale for the 90-day threshold was based on the reported length of symptoms resolution of 4 weeks (27) hence we hypothesized that in most subjects, the brain anatomy, without a second concussion, should be back to its normal stage after 90 days. Statistically significant associations (*p*-value < 0.01) between gender-age-height and each feature were mitigated by the residual method: fitting linear regression models of the reference group, and subtracting the estimated association to each feature (28). Once associations were modeled, the difference between the gender-age-height predicted value and the feature was used. Once each feature was adjusted for gender age and height, we proceeded to standardize each feature. The standardization procedure consisted of computing the median, and the IQR range of the reference group. The standardized value consisted of subtracting the median and dividing it by the IQR. After that, we proceed to compute the univariate association to the concussion status. This association was computed using the Mann–Whitney *U*-test and reported for all mean and max features. The test results of the ML steps were analyzed estimating the sensitivity, specificity, and diagnostic odds ratio from the resulting confusion matrices. The predicted probability of the ML engine was analyzed using ROC analysis and the Area under the curve (AUC). The test results of sets 2, 3, 4, and 5 were ensembled to get an estimation of a PCS index:


(4) $$\begin{array}{c} PCS\_index=\log\left(\frac{p(PCS)}{1-p(PCS)}\right), \end{array}$$

where *p*(*PCS*) is the ensembled probability of a subject affected by PCS.

The index was analyzed for associations to the number of subject concussions and the days from injury on subjects with <4 concussions. We used the spearman regression test to compute the *p*-values of trends between concussion index and the number of concussions and days from injury. We explored gender differences by running the analysis on both males and females.

## Results

### Study Subjects

Four subjects had to be removed for lack of complete clinical data. Twenty MRI data sets had severe artifacts due to the use of a defective head-neck coil where the neck receiver induced artifacts, and 11 subjects were older than 29 years, hence we removed them from the set. None of the subjects had signal abnormalities in the brain including sites of hemorrhage using the standard conventional (non-DTI) MR imaging sequences. Table 2 shows the characteristics of the 118 included in this study separated by the control and concussed subjects. Among the concussed, the PSS severity scores were similar between males and females, but the days between injury and MRI scan were larger in females than males (*p* < 0.05). Table 3 shows the distribution of the history of concussions as well as the activity involved in the last mTBI event. The main source of injuries for males was football, while soccer was the most common activity in female athletes. The concussed group was statistically significantly younger than control subjects: 15.86 (3.9) vs. 21 (2.0) (*p* < 0.001). The characteristics of the selected training cases are described in Table 4. There was no statistical difference between training cases and controls for gender, height, and weight. However, age was still statistically different between training cases and controls (*p* = 0.002).

**Table 2:** Patient characteristics.

		**Demographics**			**Injury**	
		**Age**	**Height (cm)**	**Weight (kg)**	**Days from injury**	**PSS**
Cases	Males (*n* = 51)	17 (3.8)	174 (11.9)	76 (20.8)	91 (119.0)*	36 (28)
	Females (*n* = 40)	18 (4.0)	164 (7.9)	65 (14.2)	136 (168.1)	26 (22)
Control	Males (*n* = 15)	22 (2.8)	181 (9.4)	80 (11.6)	NA	NA
	Females (*n* = 12)	20 (1.3)	165 (6.3)	63 (7.0)	NA	NA

* *p < 0.05 for statistical differences between males and females*.

**Table 3:** Concussion history and sport/injury activity of last concussion.

	**History of concussions**					**Sport activity**					
	**1**	**2**	**3**	**4**	**>5**	**Football**	**Soccer**	**Hockey ice/field**	**Lacrosse**	**Basketball**	**Not reported**
Males (*n* = 51)	10	10	11	16	4	14	7	5	4	4	17
Females (*n* = 40)	12	8	8	10	2	0	9	7	3	3	18

**Table 4:** Training set used to generate all predictive models.

	**Control**		**Training cases**	
	**(*****n*** **= 27)**		**(*****n*** **= 29)**	
	**Males**	**Females**	**Males**	**Females**
	**(*n* = 15)**	**(*n* = 12)**	**(*n* = 16)**	**(*n* = 13)**
Age	22 (2.8)	20 (1.3)	18 (3.2)	19 (4.2)
Height (cm)	181 (9.4)	165 (6.3)	181 (4.1)	166 (5.5)
Weight (kg)	80 (11.6)	63 (7.0)	83 (9.7)	71 (17.9)
Days from Injury			79 (78.0)	110 (68.1)
1 Concussion			3	5
2 Concussions			8	4
3 Concussions			5	4

### Machine Learning

**Figure 4:**
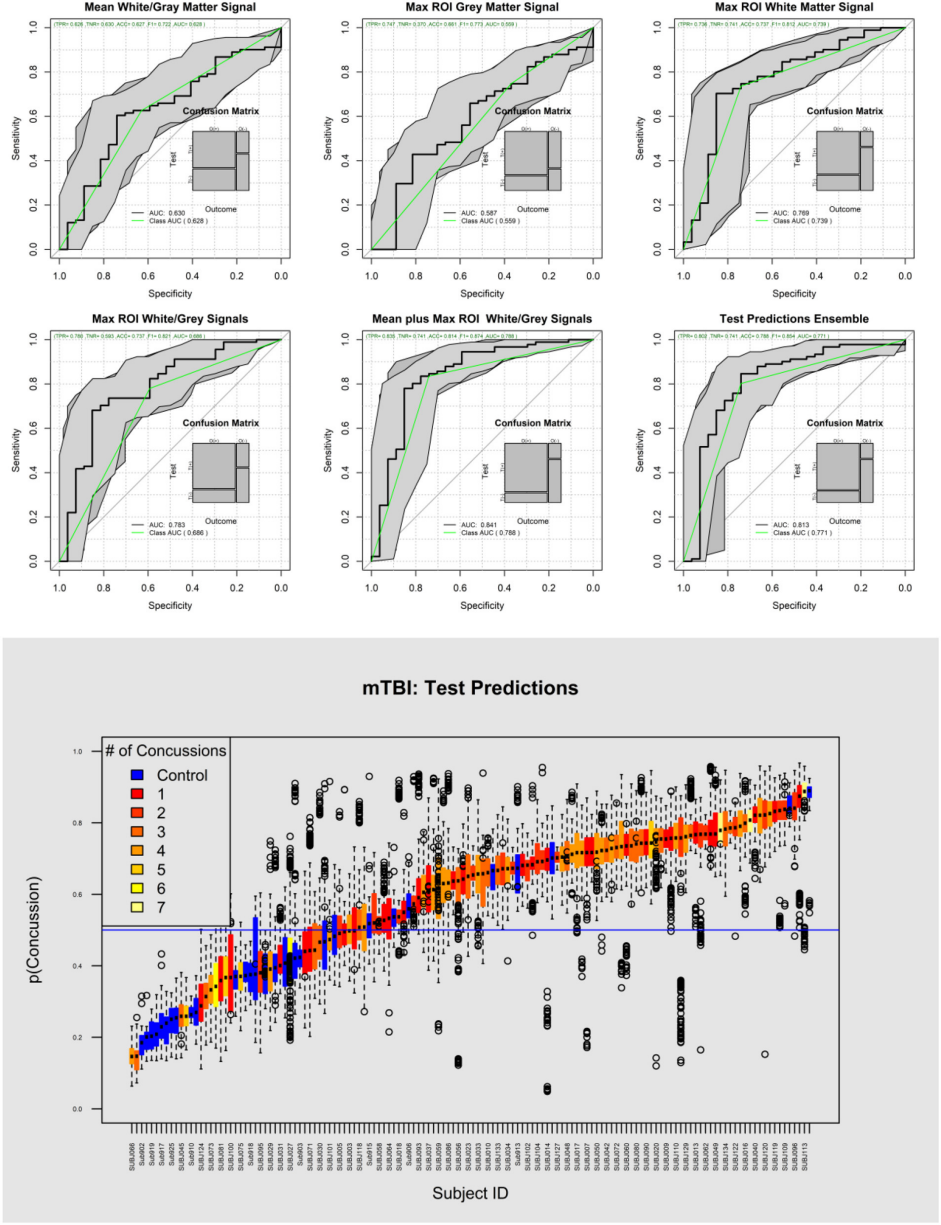
Top, ROC curves of the five different sets of analyzed data plus the average prediction performance (Ensemble prediction of the top four sets). The model based on only gray matter features poorly predicted PCS (AUC = 0.59). On the other hand, the white matter model showed superior performance (AUC = 0.77). Bottom, the probability of detecting the presence of concussion for each subject and each one of 1,000 test results are represented as box plots.

**Figure 5:**
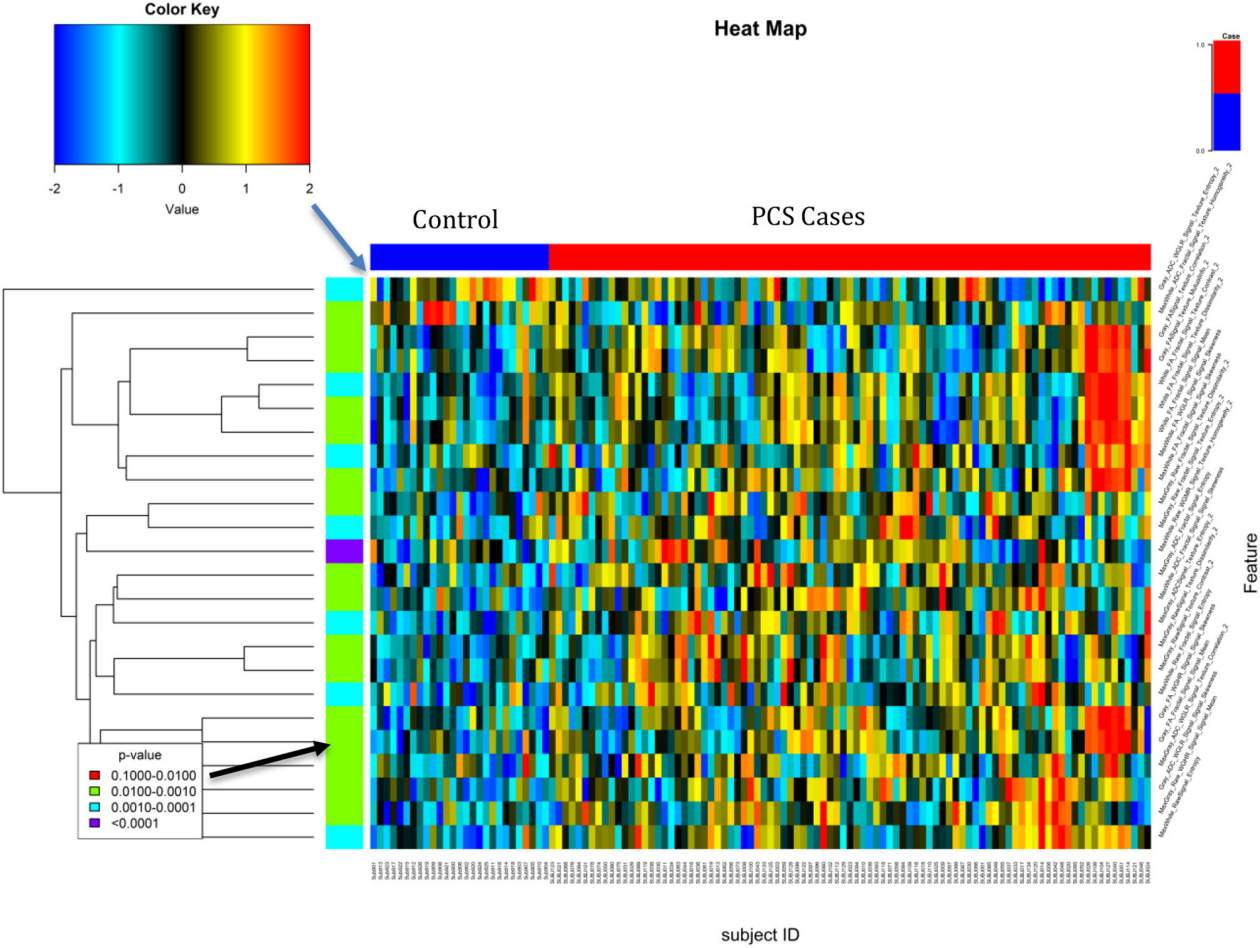
Heat map of the top features associated with PCS. The top feature was the White Matter Homogeneity maximum deviation of the lobes Max from the second level Wavelet decomposition and the MPRAGE.

Figure 4 shows the ROC plots of the five ML modeling experiments and ensemble predictions. Table 5 shows the performance of the five-set as well the ensembled predictions, and Table 6 shows the confusion matrix and the performance of the ensembled predictions. The top predictive model was based on feature set 5 (ROC AUC = 0.84), while models based on feature set 2 were at the bottom (ROC AUC = 0.59). The ensembled prediction had a ROC ACU of 0.81 (95% CI 0.71–0.92), a mean testing sensitivity of 0.81 (95% CI 0.71–0.92) with a diagnostic odds ratio of 11.6 (95% CI 4.25–31.6). The bottom plot of Figure 5 shows a color-coded boxplot per subject of the 1,000 ensembled predicted probabilities of a subject affected by PCS, where the color indicates the history of concussion for each subject.

**Table 5:** Performance of analyzed PCS models.

	**Entire brain**	**Max ROI**		**Max ROI/entire brain**		**Prediction ensemble**
	**Gray and white**	**Gray matter**	**White matter**	**MAX gray and white**	**All features**	**Max-based models**
AUC	0.63 (0.51–0.75)	0.59 (0.46–0.71)	0.77 (0.66–0.88)	0.78 (0.68–0.89)	0.84 (0.74–0.94)	0.81 (0.71–0.92)
Sensitivity	0.63 (0.52–0.73)	0.75 (0.65–0.83)	0.74 (0.63–0.82)	0.78 (0.68–0.86)	0.84 (0.74–0.91)	0.80 (0.71–0.88)
Specificity	0.63 (0.42–0.81)	0.37 (0.19–0.58)	0.74 (0.54–0.89)	0.59 (0.39–0.78)	0.74 (0.54–0.89)	0.74 (0.54–0.89)

**Table 6:** Ensemble performance of ML-based PCS diagnostic model.

	**Ensemble prediction**		
	**Cases**	**Control**	**Total**
Test+	73	7	80
Test-	18	20	38
Total	91	27	118
	**Mean**	**95% confidence intervals**	
		**2.5%**	**97.5%**
Sensitivity	0.80	0.71	0.88
Specificity	0.74	0.54	0.89
Accuracy	0.79	0.70	0.86
Diag. OR	11.6	4.25	31.6

Table 7 shows the descriptive characteristics and univariate analysis of the top significant features of the top model based on Max Gray/White and global Gray/White features (Set 5), while Figure 5 shows the heat map of the top selected features. The top feature associated with a concussion was observed as the maximum white matter homogeneity of the second level wavelet decomposition of the MPRAGE image (AUC = 0.79). Features from other image transformations and DTI maps were also highly associated with the presence of concussions. Univariate AUC ranged from 0.70 to 0.79 for the top selected features.

**Table 7:** Top features associated with PCS.

**Tissue**	**VOI**	**MRI**	**Processing**	**Feature**	**Case mean (Sd)**	**Control mean (Sd)**	**Selection Freq**	**ROC AUC**	**Wilcox *p*-value**
White	Lobe Max	MPRAGE	Wavelet (2)	Homogeneity	0.884 (0.257)	0.611 (0.249)	200%	0.792	6.41E-05
Gray	Entire	DTI-ADC	Wavelet (3)	Texture Entropy	−0.599 (0.688)	0.117 (0.823)	200%	0.769	2.67E-04
White	Lobe Max	MPRAGE	Fractal	Entropy	1.427 (0.483)	1.035 (0.490)	200%	0.764	7.65E-04
White	Lobe Max	MPRAGE	None	Entropy	1.027 (0.547)	0.618 (0.414)	200%	0.761	3.83E-04
Gray	Lobe Max	DTI-ADC	None	Texture Entropy	1.004 (0.371)	0.689 (0.251)	200%	0.748	5.44E-04
Gray	Lobe Max	MPRAGE	Fractal	Texture Entropy	1.165 (0.378)	0.818 (0.377)	197%	0.745	7.23E-04
Gray	Lobe Max	MPRAGE	Wavelet (1)	Mean	0.787 (0.317)	0.537 (0.244)	172%	0.745	1.07E-03
Gray	Entire	DTI-ADC	Wavelet (3)	Skewness	0.410 (0.727)	−0.195 (0.605)	145%	0.743	1.01E-03
White	Entire	DTI-FA	Fractal	Contrast	0.868 (1.426)	−0.246 (1.089)	200%	0.742	6.10E-04
White	Lobe Max	DTI-FA	Wavelet (3)	Skewness	1.762 (0.741)	1.199 (0.395)	200%	0.741	6.83E-04
Gray	Entire	DTI-FA	None	Correlation	1.022 (1.589)	−0.150 (1.173)	121%	0.724	1.55E-03
Gray	Entire	DTI-FA	Fractal	Mean	0.917 (1.460)	−0.152 (0.921)	127%	0.722	1.68E-03
Gray	Entire	DTI-FA	Wavelet (1)	Skewness	0.773 (1.622)	−0.335 (0.872)	114%	0.705	2.41E-03

### Statistical Analysis of the PCS Index

**Figure 6:**
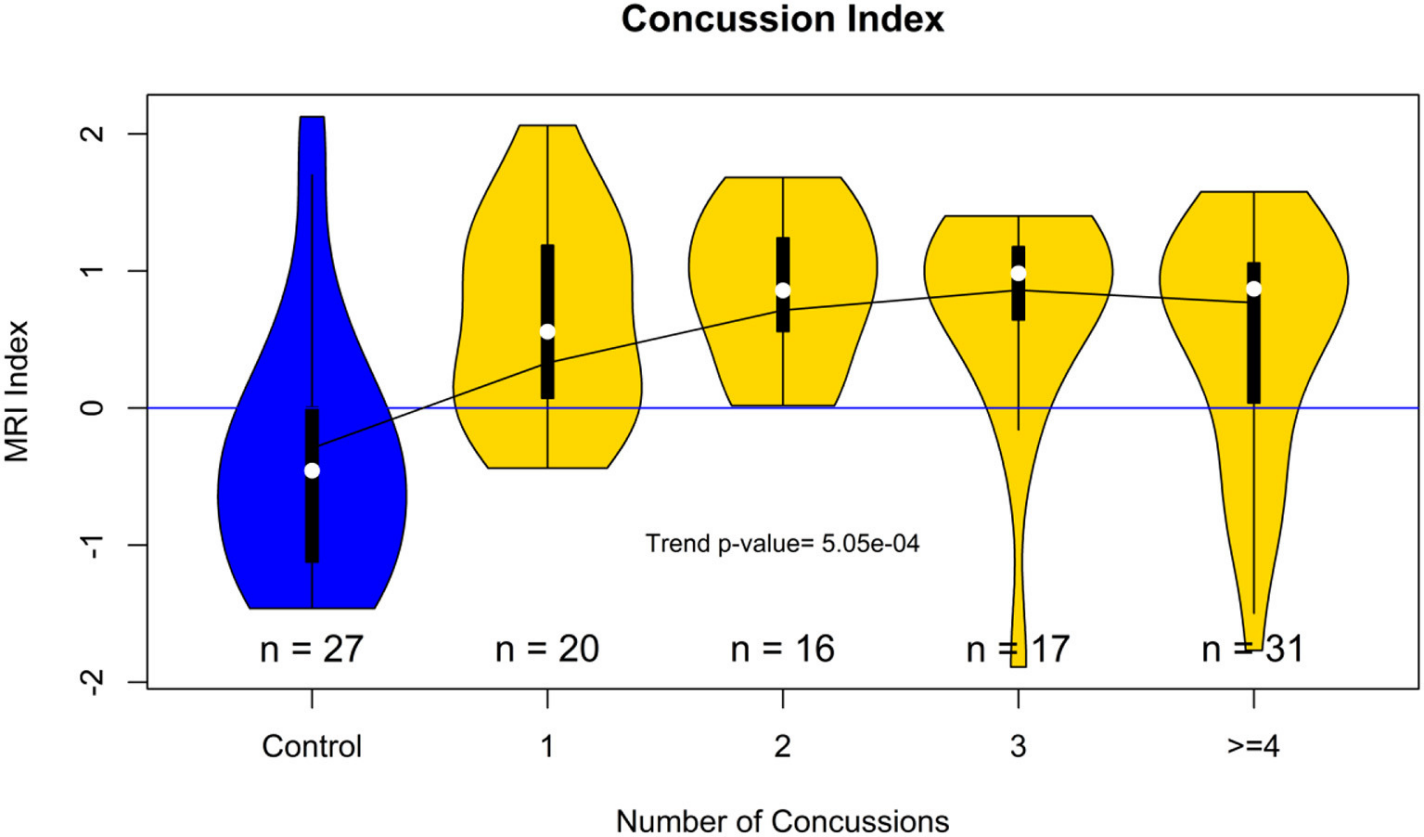
Violin plot showing the distribution of the concussion index stratified by the number of previous concussions. The plot suggests that the accumulated brain trauma plateaus after more than two concussions.

**Figure 7:**
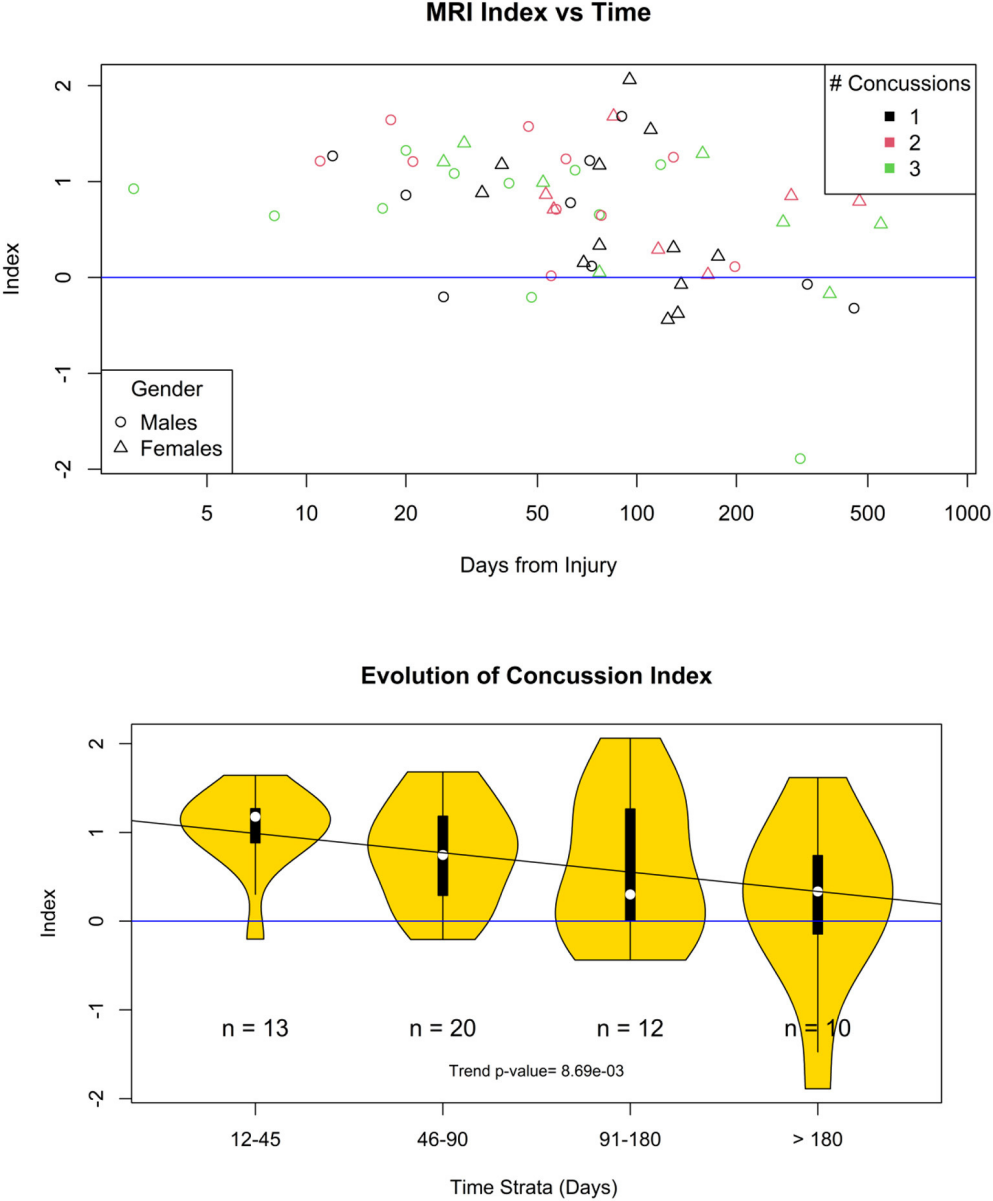
Evolution of the concussion index. Top, scatter plot of the concussion index of each one of the case participants with <4 concussions. Bottom, the time from injury was divided into four different time strata. The probability that the concuss index is not trending down is 0.009.

**Figure 8:**
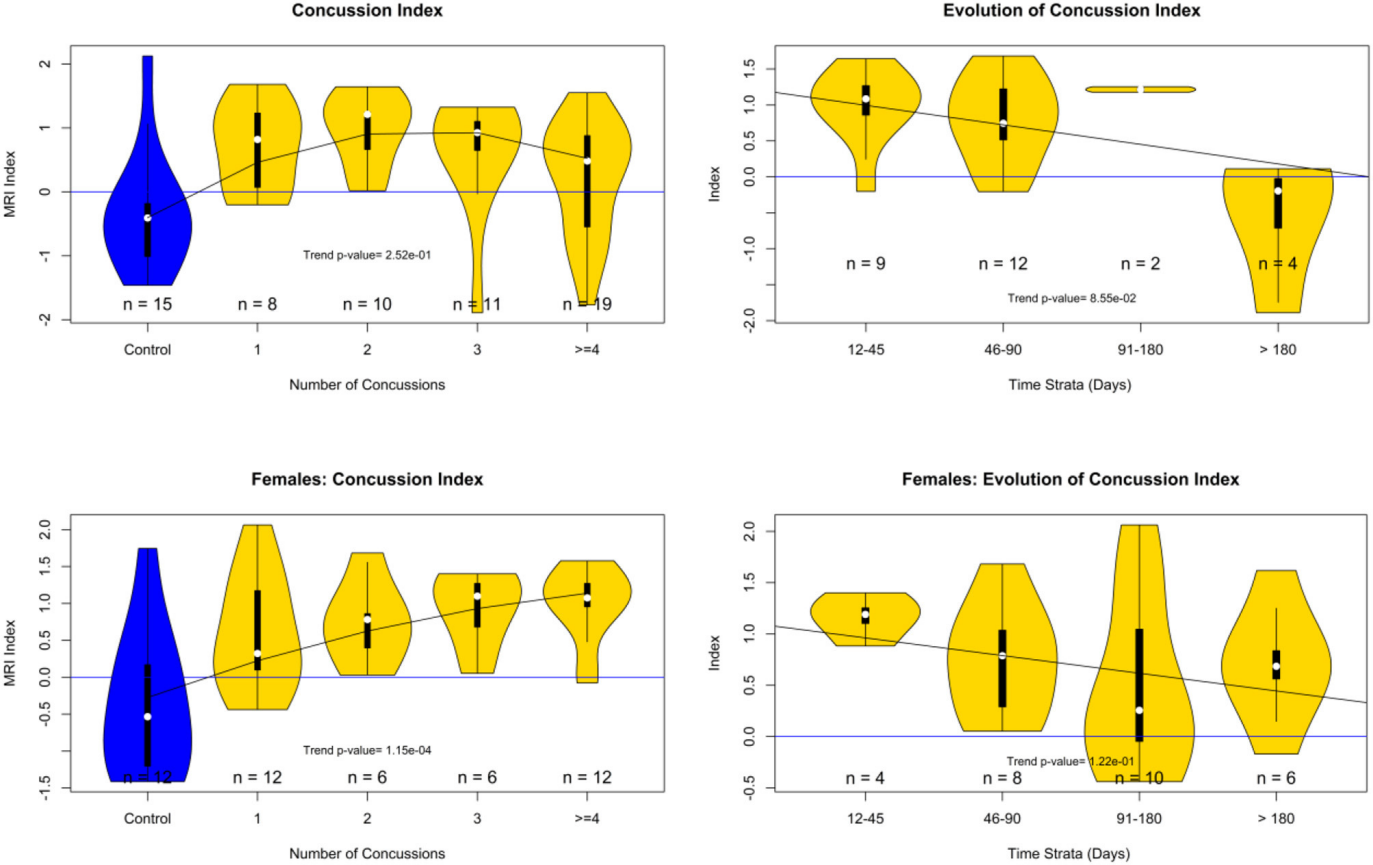
PCS Index analysis by gender. Top row males. Bottom row females. Left index value stratified by the number of concussions. Right index change by time strata (days from injury).

Figure 6 shows violin plots of the subjects stratified by the number of concussions. The trend for the association to the number of concussions was very strong (*p* < 0.001). Figure 7 shows the strength of the index over time. There was a significant reduction of the probability of detecting PCS on the subjects as the time from injury increases (*p* < 0.009). Figure 8 shows the trend analysis stratified by Gender. It is worth noting that males and females behave differently. Females showed a clear trending association with the number of previous concussions (*p* < 0.001), while males did not (*p* = 0.25). Regarding time from injury, the PCS gender stratified index was not statistically associated with days from injury for females (*p* = 0.22) nor males (*p* = 0.09).

## Discussion

This proof of principle study showcased the potential of radiomics/ML in the understanding and diagnosis of PCS from MRI series, supporting the findings of DTI and DWI studies that indicate disruption of tract integrity and conductivity on PCS patients (12, 13, 29). Our main motivation was to test the hypothesis that an MRI-based index can be used to predict the presence of residual trauma days after a mTBI event on subjects affected by PCS. Furthermore, we aimed to use standard MRI pulse sequences and well-accepted off-the-shelf machine learning procedures. The machine learning stage developed an MRI-radiomic-based PCS index for high school and collegiate athletes who were affected by an mTBI. The index was based on signal intensity and textural features from structural and diffusivity MRI image series extracted from the white/gray matter regions of the whole brain and the main eight brain lobes. The final mTBI index ensembled predictions from four different concussion models and was able to identify subjects with a history of concussions from healthy normal subjects without concussion history with a sensitivity of 0.81, and specificity of 0.74, whose 95%CI were [0.71, 0.88] and [0.54, 0.89], respectively.

We followed a radiomics approach similar to the efforts in developing predictive and diagnostic tools for brain cancers (30) and degenerative brain diseases (31). Here we explored features from structural imaging (MPRAGE) and DTI diffusivity maps (DTI-FA and DTI-ADC). Although we did not use state-of-the-art DTI post-processing, and image segmentation software, we demonstrated that the three 3D images provided useful information for the identification of brain (gray and white) tissue and the association to PCS. The MPRAGE series was successfully used for the segmentation of gray/white tissue and the identification of the eight main lobes of the brain. But, unlike other approaches that focused on single tissue properties in their feature selection process (12, 13, 29, 32), or have used only structural MPRAGE features on mTBI patients (33), we reported the comprehensive exploration of features from DTI-FA, DTI-ADC as well as MPRAGE and their derived local fractal dimensions map, and wavelet transforms.

The feature selection process identified top-performing radiomic features from the structural MPRAGE series as well as from DTI-FA and DTI-ADC images. This finding indicates that in addition to the detectable signal intensity and the texture changes in diffusivity maps, the signal intensity behavior has also changed in MPRAGE structural images, even if unable to be visually detected. That MPRAGE finding supported previous studies showcasing the potential of signal and textural information of structural imaging in the study of other brain conditions (31, 34).

The 1,000 hold-out random test results showed consistent behavior of all the trained models. The white-matter model was superior to the gray-matter model, confirming previous findings indicating that white matter is the primary tissue affected by mTBI (10, 22, 29). Furthermore, the models based on brain lobes were far superior to models based on global brain features; this is a strong indication that the brain trauma is localized, and not globally distributed (35).

Although this material does not include follow-up imaging studies, the findings showing that subjects who were imaged weeks, months, or even over a year post-injury had abnormal findings in FA, ADC, and structural images are consistent with findings from previous studies where follow-up analysis showed long-lasting FA and ADC changes in the brain (10, 11). These findings suggest that PCS may need to be taken more seriously and that indications for return to play might need to incorporate more than patient-reported symptom resolution (36). Finally, subjects with multiple prior concussions demonstrated a clear trend toward more severe mTBI index findings. Those findings agree with reports from previous longitudinal and postmortem studies from competitive athletes indicating that an increasing number of prior concussions is associated with increased and prolonged injury burden (10, 37).

The reported findings should be contrasted with alternative PCS diagnostic tests. Premorbid health conditions have been associated with severity of symptoms (38–40) as well as blood biomarkers (41–43). Premorbid conditions have been shown to be associated with severity of PCS; where PCS is higher in those with a personal history of mood disorders (38). On the other hand, blood biomarkers have been modestly associated with mTBI (41) and used to predict long-lasting PCS (44). Hence, premorbid conditions, blood biomarkers, and imaging findings could be combined in a multimodal test to improve the assessment of severity and length of PCS using machine-learning approaches.

Regarding limitations, all subjects and controls were imaged with the same MRI scanner using the same imaging protocols which provided data harmonization but limits the assessments of clinical validity in a multicenter study. Furthermore, the study did not aim to address the hypothesis of imaging findings and their association to ongoing neural repair; hence we lack evidence that current imaging findings are associated with premorbid conditions or other neural factors. Furthermore, the reported performance is still far from being applicable in a clinical care setting that usually requires 90% accuracy; henceforth the provided method may be used to ease patient concerns and near future estimations of symptom resolution. This study has other limitations: First, the sample size is relatively small, and we did not have a large reference control group. Second, the age of the controls was slightly different from that of the study subjects; hence, the discovered PCS models may be still affected by the imperfect age match and not ready for any clinical use. Third, this study lacked longitudinal observations and was not designed to test longitudinal associations. Therefore, the observed time associations of the PCS index may be a modeling artifact, thus we can't reach strong conclusions regarding the potential of MRI signatures to monitor PCS resolution. Henceforth, these study results should be reproduced and validated in a large independent cohort with longitudinal and serial MRI observations of subjects with and without persistent symptoms. Future work must include direct comparisons to blood biomarkers and premorbid Behavioral Health conditions and their combination in a multimodal setting.

## Conclusion

This proof of principle study showed that MRI radiomic features from both diffusion and MPRAGE images were different in PCS subjects from trauma-free controls. Following an ML strategy, we modeled a classifier that was able to predict the presence of concussion on 81% of the concussed subjects with a specificity of 74%. The findings suggest that the concussion-induced abnormalities on PCS subjects are not uniformly distributed among the entire brain tissue. Furthermore, this study indicated that a signal build from these MR features accumulates with the history of previous concussions. This proof of principle but tightly controlled study has shown that PCS subjects may have localized brain abnormalities, which may be invisible to conventional radiologic observation, but are present and able to be detected with radiomic feature analysis.

## Data Availability Statement

The raw data supporting the conclusions of this article will be made available by the authors, without undue reservation.

## Ethics Statement

The studies involving human participants were reviewed and approved by University of Rochester Office for Human Subject Protection. Written informed consent to participate in this study was provided by the participants' legal guardian/next of kin.

## Author Contributions

JT-P worked on the statistical analysis, drafting the first version of the paper, and reviewing the final manuscript. SM contributed to reviewing the paper and provided all the MRI images and the IRB. PR contributed to patient recruitment. ES managed the project and reviewed the manuscript. PG processed the images and reviewed the manuscript. AV designed the pulse sequence and reviewed the manuscript. ST processed the images and reviewed the quality of the MRI images as well as the segmentation. All authors contributed to the article and approved the submitted version.

## Conflict of Interest

JT-P, ST, ES, and PG are shareholders of Qmetrics Technologies. The remaining authors declare that the research was conducted in the absence of any commercial or financial relationships that could be construed as a potential conflict of interest.

## Publisher's Note

All claims expressed in this article are solely those of the authors and do not necessarily represent those of their affiliated organizations, or those of the publisher, the editors and the reviewers. Any product that may be evaluated in this article, or claim that may be made by its manufacturer, is not guaranteed or endorsed by the publisher.

## Abbreviations

AI: artificial intelligence
ML: machine learning
mTBI: mild traumatic brain injury
SRC: sports related concussion
PCS: post-concussion syndrome
DTI: diffusion weighted imaging
FA: fractional anisotropy mapping from DTI sequences
ADC: apparent diffusion coefficient from DTI sequences.

## References

[1] Faqih MU Basuki HO. Relationship of Initial Glascow Coma Scale Score and Treatment Duration with Independency Level of Patients with Head Injury in Emergency Room Dr. R. Koesma Tuban Hospital. Adv Health Sci Res. (2017) 3:130–2. 10.2991/inc-17.2017.39

[2] Nguyen R Fiest KM McChesney J Kwon CS Jette N Frolkis AD. The international incidence of traumatic brain injury: a systematic review and meta-analysis. Can J Neurol Sci. (2016) 43:774–85. 10.1017/cjn.2016.290 27670907

[3] Wilber CG Leddy JJ Bezherano I Bromley L Edwards AE Willer BS. Rehabilitation of concussion and persistent postconcussive symptoms. Semin Neurol. (2021) 41:124–31. 10.1055/s-0041-1725134 33663005

[4] Leddy JJ Baker JG Willer B. Active rehabilitation of concussion and post-concussion syndrome. Phys Med Rehabil Clin N Am. (2016) 27:437. 10.1016/j.pmr.2015.12.003 27154855

[5] Hellewell SC Beaton CS Welton T Grieve SM. Characterizing the risk of depression following mild traumatic brain injury: a meta-analysis of the literature comparing chronic mTBI to non-mTBI populations. Front Neurol. (2020) 11:350–350. 10.3389/fneur.2020.00350 32508733 PMC7248359

[6] Hellewell SC Nguyen VPB Jayasena RN Welton T Grieve SM. Characteristic patterns of white matter tract injury in sport-related concussion: an image based meta-analysis. Neuroimage Clin. (2020) 26:102253. 10.1016/j.nicl.2020.102253 32278315 PMC7152675

[7] Kara S Crosswell H Forch K Cavadino A McGeown J Fulcher M. Less than half of patients recover within 2 weeks of injury after a sports-related mild traumatic brain injury: a 2-year prospective study. Clin J Sport Med. (2020) 30:96–101. 10.1097/JSM.0000000000000811 32132366

[8] McCrory P Meeuwisse WH Aubry M Cantu RC Dvorak J Echemendia RJ. Consensus statement on concussion in sport: the 4th international conference on concussion in sport, Zurich, November 2012. J Athl Train. (2013) 48:554–75. 10.4085/1062-6050-48.4.05 23855364 PMC3715021

[9] Asken BM DeKosky ST Clugston JR Jaffee MS Bauer RM. Diffusion tensor imaging (DTI) findings in adult civilian, military, and sport-related mild traumatic brain injury (mTBI): a systematic critical review. Brain Imaging Behav. (2018) 12:585–612. 10.1007/s11682-017-9708-9 28337734

[10] Lancaster MA Meier TB Olson DV McCrea MA Nelson LD Muftuler LT. Chronic differences in white matter integrity following sport-related concussion as measured by diffusion MRI: 6-Month follow-up. Hum Brain Mapp. (2018) 39:4276–89. 10.1002/hbm.24245 29964356 PMC6179912

[11] Churchill NW Hutchison MG Richards D Leung G Graham SJ Schweizer TA. Neuroimaging of sport concussion: persistent alterations in brain structure and function at medical clearance. Sci Rep. (2017) 7:3. 10.1038/s41598-017-07742-3 28839132 PMC5571165

[12] Vergara VM Mayer AR Damaraju E Kiehl KA Calhoun V. Detection of mild traumatic brain injury by machine learning classification using resting state functional network connectivity and fractional anisotropy. J Neurotrauma. (2017) 34:1045–53. 10.1089/neu.2016.4526 27676221 PMC5333571

[13] Hirad AA Bazarian JJ Merchant-Borna K Garcea FE Heilbronner S Paul D. A common neural signature of brain injury in concussion and subconcussion. Sci Adv. (2019) 5:aau3460. 10.1126/sciadv.aau3460 31457074 PMC6685720

[14] Holli KK Harrison L Dastidar P Waljas M Ohman J Soimakallio S. Texture analysis of corpus callosum in mild traumatic brain injury patients. In: Dossel O Schlegel WC editors, World Congress on Medical Physics and Biomedical Engineering, Vol 25, Pt 4: Image Processing, Biosignal Processing, Modelling and Simulation, Biomechanics. Munich. (2010). p. 37–40. 10.1007/978-3-642-03882-2_10

[15] Gioia G. Acute concussion evaluation (ACE). Trauma. (2006) 4:8. Available online at: https://www.parinc.com/Portals/0/Webuploads/samplerpts/ACE_%20Final%20v3.pdf

[16] Custer A Sufrinko A Elbin RJ Covassin T Collins M Kontos A. High baseline postconcussion symptom scores and concussion outcomes in athletes. J Athl Train. (2016) 51:136–41. 10.4085/1062-6050-51.2.12 26885702 PMC4852319

[17] Caldwell CB Stapleton SJ Holdsworth DW Jong RA Weiser WJ Cooke G. Characterisation of mammographic parenchymal pattern by fractal dimension. Phys Med Biol. (1990) 35:235–47. 10.1088/0031-9155/35/2/004 2315379

[18] Clarke KC. Computation of the fractal dimension of topographic surfaces using the triangular prism surface-area method. Comput Geosci. (1986) 12:713–22. 10.1016/0098-3004(86)90047-6

[19] Unser M Aldroubi A. A review of wavelets in biomedical applications. Proc IEEE. (1996) 84:626–38. 10.1109/5.48870427295638

[20] Mazziotta J Toga A Evans A Fox P Lancaster J Zilles K. A four-dimensional probabilistic atlas of the human brain. J Am Med Inform Assoc. (2001) 401–30. 10.1136/jamia.2001.0080401 11522763 PMC131040

[21] Baraldi A Parmiggiani F. An investigation of the textural characteristics associated with gray-level cooccurrence matrix statistical parameters. IEEE Trans Geosci Remote Sens. (1995) 33:293–304. 10.1109/TGRS.1995.874601027295638

[22] Narayana PA. White matter changes in patients with mild traumatic brain injury: MRI perspective. Concussion. (2017) 2:CNC35. 10.2217/cnc-2016-0028 30202576 PMC6093760

[23] Cortes C Vapnik V. Support-vector networks. Mach Learn. (1995) 20:273–97. 10.1007/BF00994018

[24] Benjamini Y Hochberg Y. Controlling the false discovery rate - a practical and powerful approach to multiple testing. J Royal Statist Soc Ser B Methodol. (1995) 57:289–300. 10.1111/j.2517-6161.1995.tb02031.x

[25] Ding C Peng H. Minimum redundancy feature selection from microarray gene expression data. J Bioinform Comput Biol. (2005) 3:185–205. 10.1142/S0219720005001004 15852500

[26] Scheifier C Greil H Hermanussen M. The association between weight, height, and head circumference reconsidered. Pediatr Res. (2017) 81:825–30. 10.1038/pr.2017.3 28068308

[27] Thomas DJ Coxe K Li HM Pommering TL Young JA Smith GA. Length of recovery from sports-related concussions in pediatric patients treated at concussion clinics. Clin J Sport Med. (2018) 28:56–63. 10.1097/JSM.0000000000000413 28085687

[28] Sanfilipo MP Benedict RHB Zivadinova R Bakshi R. Correction for intracranial volume in analysis of whole brain atrophy in multiple sclerosis: the proportion vs. residual method. Neuroimage. (2004) 22:1732–43. 10.1016/j.neuroimage.2004.03.037 15275929

[29] Mitra J Shen KK Ghose S Bourgeat P Fripp J Salvado O. Statistical machine learning to identify traumatic brain injury (TBI) from structural disconnections of white matter networks. Neuroimage. (2016) 129:247–59. 10.1016/j.neuroimage.2016.01.056 26827816

[30] Zhang S Chiang GCY Magge RS Fine HA Ramakrishna R Chang EW. Texture analysis on conventional MRI images accurately predicts early malignant transformation of low-grade gliomas. Eur Radiol. (2019) 29:2751–9. 10.1007/s00330-018-5921-1 30617484

[31] Sorensen L Igel C Pai A Balas I Anker C Lillholm M. Differential diagnosis of mild cognitive impairment and Alzheimer's disease using structural MRI cortical thickness, hippocampal shape, hippocampal texture, and volumetry. Neuroimage Clin. (2017) 13:470–82. 10.1016/j.nicl.2016.11.025 28119818 PMC5237821

[32] Lui YW Xue Y Kenul D Ge Y Grossman RI Wang Y. Classification algorithms using multiple MRI features in mild traumatic brain injury. Neurology. (2014) 83:1235–40. 10.1212/WNL.0000000000000834 25171930 PMC4180485

[33] Holli KK Harrison L Dastidar P Waljas M Liimatainen S Luukkaala T. Texture analysis of MR images of patients with mild traumatic brain injury. BMC Med Imaging. (2010) 10:8. 10.1186/1471-2342-10-8 20462439 PMC3161385

[34] Michoux N Guillet A Rommel D Mazzamuto G Sindic C Duprez T. Texture analysis of T2-weighted MR images to assess acute inflammation in brain MS lesions. PLoS ONE. (2015) 10:145497. 10.1371/journal.pone.0145497 26693908 PMC4687842

[35] Holli KK Waljas M Harrison L Liimatainen S Luukkaala T Ryymin P. Mild traumatic brain injury: tissue texture analysis correlated to neuropsychological and DTI findings. Acad Radiol. (2010) 17:1096–102. 10.1016/j.acra.2010.04.009 20605490

[36] Curry AE Arbogast KB Metzger KB Kessler RS Breiding MJ Haarbauer-Krupa J. Risk of repeat concussion among patients diagnosed at a pediatric care network. J Pediatr. (2019) 210:13. 10.1016/j.jpeds.2019.04.001 31101406 PMC6645379

[37] Henry LC Elbin RJ Collins MW Marchetti G Kontos AP. Examining recovery trajectories after sport-related concussion with a multimodal clinical assessment approach. Neurosurgery. (2016) 78:232–41. 10.1227/NEU.0000000000001041 26445375 PMC4833014

[38] Morgan CD Zuckerman SL Lee YM King L Beaird S Sills AK. Predictors of postconcussion syndrome after sports-related concussion in young athletes: a matched case-control study. J Neurosurg Pediatr. (2015) 15:589–98. 10.3171/2014.10.PEDS14356 25745949

[39] Karr JE Iverson GL Isokuortti H Kataja A Brander A Ohman J. Preexisting conditions in older adults with mild traumatic brain injuries. Brain Injury. (2021) 2021:1–9. 10.1080/02699052.2021.1976419 34546830

[40] Putukian M Riegler K Amalfe S Bruce J Echemendia R. Preinjury and postinjury factors that predict sports-related concussion and clinical recovery time. Clin J Sport Med. (2021) 31:15–22. 10.1097/JSM.0000000000000705 30540572

[41] Hier DB Obafemi-Ajayi T Thimgan MS Olbricht GR Azizi S Allen B. Blood biomarkers for mild traumatic brain injury: a selective review of unresolved issues. Biomarker Res. (2021) 9:5. 10.1186/s40364-021-00325-5 34530937 PMC8447604

[42] Shahim P Tegner Y Marklund N Blennow K Zetterberg H. Neurofilament light and tau as blood biomarkers for sports-related concussion. Neurology. (2018) 90:E1780–8. 10.1212/WNL.0000000000005518 29653990 PMC5957307

[43] Hiskens MI Schneiders AG Angoa-Perez M Vella RK Fenning AS. Blood biomarkers for assessment of mild traumatic brain injury and chronic traumatic encephalopathy. Biomarkers. (2020) 25:213–27. 10.1080/1354750X.2020.1735521 32096416

[44] Siman R Cui HM Wewerka SS Hamel L Smith DH Zwank MD. Serum SNTF, a surrogate marker of axonal injury, is prognostic for lasting brain dysfunction in mild TBI treated in the emergency department. Front Neurol. (2020) 11:249. 10.3389/fneur.2020.00249 32322237 PMC7156622

